# INVESTIGATE-I (INVasive Evaluation before Surgical Treatment of Incontinence Gives Added Therapeutic Effect?): study protocol for a mixed methods study to assess the feasibility of a future randomised controlled trial of the clinical utility of invasive urodynamic testing

**DOI:** 10.1186/1745-6215-12-169

**Published:** 2011-07-06

**Authors:** Megan Murdoch, Elaine McColl, Denise Howel, Mark Deverill, Brian S Buckley, Malcolm Lucas, Christopher R Chapple, Douglas G Tincello, Natalie Armstrong, Cath Brennand, Jing Shen, Luke Vale, Paul Hilton

**Affiliations:** 1 Directorate of Women's Services, Royal Victoria Infirmary, Newcastle upon Tyne, UK; 2 Institute of Health & Society, Newcastle University, Newcastle upon Tyne, UK; 3 Bladder & Bowel Foundation, Morriston Hospital, Swansea, UK; 4 Department of Urology, Morriston Hospital, Swansea, UK; 5 Department of Urology, The Royal Hallamshire Hospital, Sheffield, UK; 6 Department of Obstetrics & Gynaecology, University of Leicester, UK; 7 Department of Health Sciences, University of Leicester, UK

## Abstract

**Background:**

Urinary incontinence is an important health problem to the individual sufferer and to health services. Stress and stress predominant mixed urinary incontinence are increasingly managed by surgery due to advances in surgical techniques. Despite the lack of evidence for its clinical utility, most clinicians undertake invasive urodynamic testing (IUT) to confirm a functional diagnosis of urodynamic stress incontinence before offering surgery for this condition. IUT is expensive, embarrassing and uncomfortable for women and carries a small risk. Recent systematic reviews have confirmed the lack of high quality evidence of effectiveness.

The aim of this pilot study is to test the feasibility of a future definitive randomised control trial that would address whether IUT alters treatment decisions and treatment outcome in these women and would test its clinical and cost effectiveness.

**Methods/design:**

This is a mixed methods pragmatic multicentre feasibility pilot study with four components:-

(a) A multicentre, external pilot randomised trial comparing basic clinical assessment with non-invasive tests and IUT. The outcome measures are rates of recruitment, randomisation and data completion. Data will be used to estimate sample size necessary for the definitive trial.

(b) Qualitative interviews of a purposively sampled sub-set of women eligible for the pilot trial will explore willingness to participate, be randomised and their overall trial experience.

(c) A national survey of clinicians to determine their views of IUT in this context, the main outcome being their willingness to randomise patients into the definitive trial.

(d) Qualitative interviews of a purposively sampled group of these clinicians will explore whether and how they use IUT to inform their decisions.

**Discussion:**

The pilot trial will provide evidence of feasibility and acceptability and therefore inform the decision whether to proceed to the definitive trial. Results will inform the design and conduct of the definitive trial and ensure its effectiveness in achieving its research aim.

**Trial registration number:**

Current Controlled Trials ISRCTN71327395 assigned 7^th ^June 2010.

## Background

### Scientific background

Urinary incontinence (UI) can dramatically influence the physical, psychological and social wellbeing of affected individuals, their families and carers and has considerable resource implications for the health service. The estimated prevalence of UI in general population studies varies from 5% to 69% with most estimates between 25% and 45%. In middle aged and older women daily incontinence estimates vary from 5% to 15% [1]. The majority of these women have stress urinary incontinence (SUI), 50%, or mixed urinary incontinence (MUI), 36% [2]. A 2004 UK study estimated the annual cost to the National Health Service of treating clinically important UI in women at £233m [3].

Urodynamic testing is one of several methods that may be used in the assessment of UI to guide management decisions, including whether surgical intervention is indicated. This group of investigations is used to evaluate the function of the lower urinary tract and can be invasive (requiring catheterisation) or non-invasive [4]. The most commonly used invasive urodynamic test (IUT) is multi-channel cystometry;[5] this looks at the pressure/volume relationships during bladder filling and emptying, with a view to defining a functional, as distinct from symptomatic, diagnosis. Whilst this is the most commonly used IUT, videocystometry and ambulatory bladder pressure monitoring are also used. Non-invasive tests include frequency/volume charting, mid-stream urine culture, urine flow rate and residual urine volume measurement by ultrasound [6].

The current position of IUT in the diagnostic pathway is not agreed, and practice varies considerably. In a UK survey in 2002 only half of the units surveyed had guidelines on indications for the tests, and 84.5% carried out cystometry in all women with incontinence [5]. Current guidance from the National Institute for Health and Clinical Excellence (NICE) suggests that cystometry is not required prior to conservative treatments, and that there is no evidence to support its use prior to surgery where the diagnosis of SUI is likely [7]. NICE, the National Institute for Health Research - Health Technology Assessment (NIHR-HTA) programme, the Cochrane Collaboration and the International Consultations on Incontinence (ICI) have all recently undertaken systematic reviews on the subject of urodynamic testing, and all emphasize the lack of high quality primary research confirming clinical utility [4,7-10]. Patients and clinicians working together in a project associated with the James Lind Alliance (JLA) have identified IUT as a research priority and, along with NICE, have recommended further research to answer the question of whether the use of IUT, prior to initial or subsequent treatments, affects the outcomes and cost effectiveness of interventions in women with UI [7,10].

The costing report associated with the NICE clinical guideline on UI used an estimated charge of £176 for each IUT. Consequently, the annual potential saving from eliminating IUT prior to conservative treatment was estimated at approximately £3 m and savings from a more rational use of IUT before surgery for SUI at approximately £321,000 [7]. These calculations are based on 2002 -2007 figures. Changes in available operative techniques have led to dramatic alterations in surgical practice as recorded in the NHS's Hospital Episode Statistics (HES) which showed a 54% increase in surgery for SUI over the last ten years [11]. These data along with 2008-09 national tariff costs for IUT of £425 suggest that the savings from a more rational use of IUT prior to surgery are likely to be substantially greater.

A randomised controlled trial (RCT) of IUT versus less invasive procedures prior to surgery would provide gold-standard evidence of the clinical and cost-effectiveness of this expensive procedure. However, prior to proceeding to a full scale RCT, a feasibility pilot study is deemed necessary for several reasons;

The use of IUT is widely established in clinical practice; many experts consider it a mandatory investigation, particularly prior to surgery [12,13]. So we need to establish whether sufficient clinicians are in equipoise and willing to recruit and randomise patients to a RCT. There is growing evidence in favour of pilot studies involving collection of both quantitative and qualitative data prior to embarking on this type of definitive RCT [14].

Patients may not see the importance of 'testing a test' and are usually willing to undergo even invasive investigations in the belief that they guide their clinician and themselves to the most appropriate management. Trials reported in 2000 and 2001 randomizing radiography investigations for low back pain were only able to recruit 23% and 51% of patients approached to enter the randomised arms [15,16]. By assessing patients' willingness to undergo randomisation, potential barriers and facilitators to participation can be identified and used to modify the design of the definitive trial.

Given these uncertainties, before carrying out a definitive trial, it is essential to undertake a pilot study to estimate the extent of the potential recruitment problems described above and to provide robust estimates of the variability of the proposed outcome measures and of the likely rates of eligibility, recruitment, randomisation and retention of participants.

## Specific objectives

### The future definitive trial (INVESTIGATE II)

The objective of the proposed future definitive trial is to address the question of whether IUT compared to basic clinical assessment (non-invasive) alters treatment decisions and outcomes, including the incremental cost per quality adjusted life year (QALY) gained, in women potentially suitable for surgical treatment of SUI or stress predominant MUI. The outcome measures will include quantified post-treatment urinary leakage, impact on general health and condition-specific quality of life, adverse effects from investigation or treatment, and health economic outcomes. The latter will include mean incremental cost, mean incremental QALYs, and incremental cost per QALY. Thus, in the definitive trial, we hope to establish whether IUT should indeed be offered to all women prior to surgery.

### The pilot trial (INVESTIGATE I)

The objective of this pilot trial is to test the logistics and feasibility of the definitive trial, specifically;

● To carry out an external (rehearsal) pilot trial randomising patients between basic assessment and IUT to assess patient recruitment and willingness to be randomised, rehearse methodology, and provide outcome data to inform sample size calculations for a definitive trial.

● To explore women's reasons for participation or non-participation and their experiences of the pilot trial procedures. Their responses may help not only in the evaluation of the feasibility of the definitive trial, but also in the planning and optimising of future recruitment and retention.

● To assess clinicians' views on IUT in this particular context and their willingness to randomise patients within a definitive trial.

## Methods/Design

### Trial design

The pilot feasibility study is a mixed methods pragmatic multicentre study [17] with four components, conducted over a two year period, as follows:-

1. A multicentre randomised external pilot trial comparing basic clinical assessment and IUT. The design fully rehearses the research design and outcome measures proposed for the definitive RCT.

2. Qualitative interviews with a purposely sampled subset of women eligible for the trial to explore their reasons for agreeing (or not) to participate, their willingness to be randomised, and their experiences of the pilot trial.

3. A national web-based survey of relevant clinicians' views about urodynamics and their willingness to enter their patients into the definitive trial; the membership lists of the appropriate professional bodies and special interest groups will be used as the sampling frame.

4. Qualitative interviews with a small subset of clinicians (respondents to the survey) to explore whether and how they use the results of IUT to inform their decisions, and to contextualise the questionnaire responses.

## Randomised trial

### Participants

Women will be recruited from six UK units which include a mix of specialist urogynaecology (Newcastle upon Tyne Hospitals NHS Foundation Trust & University Hospitals of Leicester NHS Trust), female urology (Morriston Hospital, Swansea & Royal Hallamshire Hospital, Sheffield) and general gynaecology (Queen Elizabeth Hospital, Gateshead & Wansbeck Hospital, Northumberland) departments. These units provide secondary +/- tertiary level of care and are representative of the UK units who would be invited to participate in the definitive trial. Eligibility criteria for the pilot trial are those anticipated for the future definitive trial.

#### Inclusion criteria

Women fulfilling the following characteristics

● With a clinical diagnosis of SUI or stress predominant MUI

● Who consider that their family is complete

● Who have undergone a course of pelvic floor muscle training (+/- other non surgical treatments for their urge symptoms) with inadequate resolution of symptoms

● Both the woman herself and her treating clinician should agree that surgery is an appropriate and acceptable next line of treatment.

#### Exclusion criteria

● Symptomatic utero-vaginal prolapse requiring treatment

● Previous surgery for urinary incontinence or pelvic organ prolapse

● Urodynamic investigation within the last three years

● Neurological disease causing urinary incontinence

● Current involvement in competing research studies

● Unable or unwilling to give informed consent

Over a nine-month recruitment period, assigned research nurses will identify and obtain consent from eligible patients. The outpatient clinics run by each unit will be the source of potential recruits. Notes will be screened prior to the appointment and a patient information sheet posted with the appointment letter if, in so far as can be judged from records alone, the patient meets eligibility criteria. This will allow any questions that the woman may have to be addressed within the forthcoming clinic visit. Informed, written consent will be taken from those who agree to take part.

A small number may be identified only at the time of attendance at the clinic. They will be invited to participate and given the relevant information. The research nurse will contact the patient after a period of at least 24 hours, respond to any questions they may have and review their decision regarding involvement.

Consent to retain contact details will be obtained from those declining recruitment into the pilot trial but who are willing to be contacted for the qualitative interviews.

Participants will be informed prior to recruitment that they can withdraw at any stage of the research without having to provide a reason and that withdrawal will not affect the care they receive.

### Randomisation

Patients who have given consent will be randomised 1:1 to either the control or intervention arm, by an internet-accessed system operated by the Newcastle Clinical Trials Unit. Randomisation will be stratified by centre, using a random block length to minimise the risk of breech of concealment of allocation.

### Blinding

Due to the nature of this trial it is not possible to blind participants and clinicians after randomisation.

### Intervention and control conditions

#### Control arm

Participants will undergo basic clinical assessment supplemented by non-invasive tests as directed by the clinician. These may include frequency/volume charting, mid-stream urine culture, urine flow rate and residual urine volume measurement by ultrasound; they will not undergo invasive urodynamic testing. Subsequently, if agreed to by the clinician and the patient, they will undergo surgical treatment. Given the pragmatic nature of the study the choice of operation will be left to the individual surgeon and patient.

#### Intervention arm

Participants will undergo assessment as above and will also undergo invasive urodynamic testing. Usually this will be multi-channel cystometry; however, videourodynamics and long-term ambulatory bladder pressure monitoring may also be included at the discretion of the clinician.

Women will undergo similar surgical treatment if urodynamic stress incontinence (USI) is confirmed on IUT. This will comprise the majority of women. Where other diagnoses are identified they will be offered alternative treatments according to current NICE recommendations.^5 ^In some cases, where mixed abnormalities are reported, women may first undergo one or more of these treatments and then proceed to surgery for SUI. Postoperative follow up will be arranged in accordance with the clinician's usual practice.

### Data collection

The questionnaires to be administered in the pilot trial are those proposed for the definitive trial, as described below.

Following randomisation all patients will be asked to complete baseline study outcome questionnaires.

Data will be collected from the hospital records throughout the patient's participation in the study to rehearse the data collection methods for the definitive trial. Data will include details of non-invasive and invasive investigations, operative and in-patient procedures including postoperative complications, non-surgical treatments and resource utilisation.

Women in both arms will complete a follow-up study outcome questionnaire six months after the first treatment they receive in the trial. Questionnaire response will be encouraged by providing prepaid envelopes and posting one-month reminder letters.

### Outcomes

#### The pilot trial

The outcome measures for the initial feasibility study are:

● Number of eligible patients in each unit, and the rates of recruitment, randomisation and data completion.

● The confirmation or otherwise that units are able to identify the required number of eligible women and recruit them.

● The acceptability of the investigation strategies as manifest though recruitment and retention levels.

● The feasibility and acceptability of data collection tools measured by completion rates and quality of data.

● Clinical outcome data to estimate the necessary sample size for the definitive trial.

Whilst it is not possible to be categorical about it at this stage, the criteria used to establish feasibility, and to confirm a decision to proceed with a definitive trial, will be determined as a compound function of the above factors.

The required sample size for a subsequent definitive trial will be calculated using the clinical outcome data from the pilot trial. A definitive trial will be judged to be feasible if the numbers of eligible patients recruited, randomised and retained within the pilot trial, plus the number of surgeons in clinical equipoise on the subject who are prepared to randomise their patients indicate that it will be possible to generate the required sample within a period of 24 months.

The protocol for the definitive trial will be modified to improve recruitment based upon the findings of the qualitative interviews with patients not randomised to the pilot trial. Similarly, if the experiences of those women randomised, or the study team's experience of the pilot indicate that protocol modifications may be of value then these would also be considered within the judgment of feasibility.

#### The definitive trial

Should we proceed to a definitive trial, the currently favoured primary outcome is the combined symptom score of the International Consultation on Incontinence Female Lower Urinary Tract Symptoms questionnaire (ICIQ-FLUTS) at 6 months after treatment [18]. A patient reported outcome measure has been selected because clinician assessment of patient outcomes tends to underestimate the patients' perceived symptoms [19].

Secondary outcomes will include the quantification of urinary leakage (three day bladder diary and ICIQ-UI Short Form), the prevalence of symptomatic '*de novo' *functional abnormalities including voiding dysfunction and detrusor overactivity (subscales of ICIQ-FLUTS, with cystometry in symptomatic patients), the impact of urinary symptoms on quality of life (ICIQ-LUTSqol and Urogenital Distress Inventory (UDI)) and utility values from the EQ-5D questionnaire and SF-6D (utility score generated from SF-12 questionnaire) [20]. These are rehearsed within the pilot study with a view to refining the choice and timing of outcomes for the main trial, based on data yield (*e.g*. the percentage of recruited participants returning completed questionnaires) and quality (*e.g*. the completeness and consistency of responses within returned questionnaires).

### Sample size

There is relatively little information on the distribution of the ICIQ-FLUTS combined symptom score or on recruitment, randomisation and retention rates in the relevant patient population. As a result there are insufficient data to calculate the sample size required in a definitive trial to yield adequate power to detect a clinically important difference in the primary outcome measure. This is one of the main reasons to undertake the pilot trial.

The sample size for pilot trials is typically determined pragmatically, with recommendations of a minimum of 30 participants per arm [21]. We aim to recruit 60 per trial arm to investigate the distribution of the outcome measures. Previous trials in the area of pelvic floor dysfunction, including investigation,[22] surgical,[23-25] and non-surgical treatments [26] suggest attrition rates of 13% (7-20%) between identification and randomization, 16% (6-20%) between randomization and treatment, and 13% (9-20%) between treatment and follow-up at 6 months. Taking the more pessimistic figure in each case we estimate that a total of 240 eligible patients should be approached allowing for a 50% overall attrition. The recruiting units collectively undertake 540 relevant procedures per year; therefore, identifying 240 eligible women within the nine month recruitment period should not present undue difficulty.

### Results/analysis

Data will be analysed after all patients have completed six months follow up. The statistical analysis will be descriptive in nature and provide estimates of key trial variables for the definitive trial to inform power calculations and other aspects of the trial design. The cost-utility analysis will be rehearsed which may inform the study hypothesis for the definitive trial as well as informing the analysis plan.

## Qualitative interviews with women eligible for the randomised trial

### Participants

A sample of women approached to participate in the pilot trial will be invited to take part in the qualitative interviews. Purposive sampling will be used to include women from a range of ages, trial participation status (did not agree to participate, agreed to participation and retained to follow-up, agreed to participation but provided incomplete follow up data), allocation status (IUT or basic assessment), treatment received (surgery or conservative), and study site.

### Intervention

The selected women will receive a postal invitation from the Newcastle Clinical Trials Unit. If they are interested in participating, they will be asked to return an enclosed expression of interest form. Arrangements for the interview will then be made. Women declining participation into the pilot trial will be approached as soon as possible after that decision; those participating in the trial will be approached at the end of their participation in the trial to capture their overall experience.

Written, informed consent will be taken prior to the interview. An expert qualitative interviewer will conduct the interviews which, with permission, will be audio-recorded and transcribed verbatim. Interviews will be semi-structured using a prompt guide with broad topic areas but emphasis will be on discussing the women's own perspectives freely. The prompt guide has been developed using a combination of the background literature and expert qualitative research experience. It may be modified, if necessary, as the interviews progress to incorporate issues raised by interviewees that have not been anticipated but are of interest [27]. It is anticipated that 25-30 interviews will be required, but data collection will continue until a point of theoretical saturation has been reached [28,29].

### Results/Analysis

The aims of the interviews are to explore women's understanding and experience of the study, their decisions around participation and their perceived barriers to and facilitators of participation.

Their responses may help not only in the evaluation of the feasibility of a definitive trial, but also in the planning and optimising of recruitment and retention e.g. to refine the content of information, recruitment and data collection procedures used.

Both data collection and analysis will be iterative. Data will be analysed using the constant comparative method,[29] supported by NVivo software [30].

## National clinician survey

### Participants

All members of the British Society of Urogynaecology (BSUG) and British Association of Urological Surgeons Section of Female, Neurological and Urodynamic Urology (BAUS-SFNUU) with current email addresses on the membership database of the respective organisations will be involved. Apart from a few exceptions, clinicians are only members of one society. These urologists and gynaecologists are the intended source of patient recruitment for the definitive trial and reviews indicate that endorsement by respected authorities is a factor in enhancing response rates in postal and self-completion surveys [31-34].

### Intervention

Members will be invited by email, sent from the society secretariat, with the support of the research committees of the aforementioned societies, to complete either a web-based ('http://SurveyMonkey.com') or paper-based questionnaire; this support has already been obtained from both societies. Reminders will be sent three and six weeks after initial contact to encourage and stimulate response [31,32,34]. The data set will be closed and prepared for analysis twelve weeks after initial contact as experience in previous surveys shows that the majority of responses are made within this period. This survey approach will enable collection of data from a large sample of respondents over a wide geographical area in a timely and efficient manner.

The survey questionnaire will contain both open and closed questions and will ask for the respondent's views about, access to, and current use of IUT. Part of the survey will include brief details of the proposed definitive trial in the form of a 'vignette' and will seek to ascertain the clinician's willingness to participate and randomise their patients within such a trial. The questionnaire will be piloted on non-members to assess comprehensibility and content validity.

### Results/analysis

The method of statistical analysis will be descriptive in nature. Data will be collected on the strength of surgeons' views for the necessity of invasive dynamic testing for a set of vignettes using an 11-point Likert scale. These distributions will be summarised by their medians, quartiles and range. Other variables such as their willingness to take part in a future randomised trial and views on the importance of the questions will be summarised by percentages in each category.

## Qualitative clinician interviews

### Participants/intervention/analysis

Clinicians participating in the national survey will be invited, at the same time, to take part in this interview. If they accept they will be asked to return an expression of interest form with their contact details.

A purposive sample of these clinicians will undergo a short telephone interview to explore their interpretation of IUT and how they use the results to decide on the most appropriate treatment options. Twelve interviews should ensure that a range of views are represented (e.g. those who do and do not currently use IUT, those who do and do not feel is it an important part of their decision making and those who would or would not be willing to participate in a later trial), but data collection will continue until a point of theoretical saturation is reached [27].

Telephone interviews enable this to be done efficiently. Electronic, written consent will be obtained prior to the interview.

Analysis will identify key themes related to the use of IUT and possible involvement in a later trial.

## Trial governance

A favourable ethical opinion for this research has been received from Newcastle and North Tyneside No1 Research Ethics committee (reference10/H0906/76).

The operational management of the trial will be overseen by a Trial Management Group that has responsibility for ensuring the compliance and progress of the study in relation to all regulatory, administrative, academic and clinical/safety issues.

The Data Monitoring and Ethics Committee (DMEC) will focus on the safety and ethical issues. Their role will be to monitor data and make recommendations on whether or not the trial should continue for ethical or safety reasons. DMEC membership will comprise an independent chair, an independent statistician and one other member, independent of the research team, with relevant content area or methodological expertise.

The Trial Steering Committee (TSC) will provide overall supervision for the trial on behalf of the Trial Sponsor (Newcastle upon Tyne Hospital NHS Foundation Trust) and Trial Funder (NIHR HTA) and will ensure that the trial is conducted in accordance with the principles of Good Clinical Practice [35]. The committee membership comprises and independent chair, two other independent members with relevant content area or methodological expertise, two lay members, the chief investigator, another principal investgator, the trial statistician, and the senior trial manager.

R&D approval for all participating sites has been sought, via the NIHR Coordinated System for gaining NHS permissions (CSP); this system seeks to ensure that all quality assurance and statutory requirements in respect of clinical research are met, through standardising and streamlining the process for gaining NHS Permission in England [36].

All data will be kept in accordance with Caldicott Principles,[37] and will be archived at the Newcastle Upon Tyne Hospitals NHS Foundation Trust archive facility for ten years following the last publication from the study.

## Discussion

Pilot studies play an important role in health research. A well-conducted pilot study with clear aims and objectives encourages methodological rigour, ensures that the work is scientifically valid and publishable, and results in higher quality RCTs [21]. Lancaster et al. produced a framework and addressed some of the methodological reasons for undertaking pilot studies related to RCTs [21]. Using this framework as a guide we summarise below our justifications for undertaking a pilot study and its methodology.

The randomised external pilot trial rehearses the methodology proposed for the definitive RCT. A run through of the trial procedures on a smaller scale will practise and identify any logistical issues that might be modified. The number of eligible patients and rates of recruitment and randomisation will confirm whether the required sample size for a definitive trial could be identified and recruited. Recruitment has implications for both timing and funding of a definitive trial and poor recruitment is one of the major reasons for abandoning trials early [21,38]. A RCT to address whether IUT alters treatment decisions and treatment outcome in women suitable for surgical treatment of SUI or stress predominant MUI is likely to require large numbers of participants and would therefore be expensive and time-consuming. Prior to allocating resources to such a large trial feasibility must be determined to reduce the risk of the definitive trial not being able to recruit to time and target.

Data collection for the proposed definitive trial consists of both clinical records data and patient self-completed questionnaires. The outcome questionnaires rely on patient recall over a six month period and analysis of completion rates and quality of data will not only test comprehensibility but guide timing of future data collection. Several secondary outcome measures have been chosen with a view to refining the choice on the basis of data yield and quality. The demands of a trial are an identified barrier to patient participation and refinement of the data collection tools will ensure these are kept to a minimum [38].

Qualitative interviews with the women will explore their reasons for participating, or not, and their experiences of the trial with the potential benefit of guiding protocol amendments such as the consent procedure and questionnaire format, to improve the recruitment process and questionnaire responses.

The acceptability of the protocol is an important aspect of the trial design. As mentioned previously IUT is widely established in clinical practice and the absence of this investigation prior to offering surgery is a potential barrier to clinicians' willingness to randomise their patients. Clinician surveys in other urogynaecology research have identified clinicians' views of perceived benefit and complications of the two arms as impacting participation [14]. Women may also not see the benefit of testing a test, believing that the results of any available test must guide their clinician and themselves to the most appropriate management. Prior to embarking on a definitive RCT we need to ensure that not only are the participants willing to be randomised but that the clinicians are also willing to randomise their patients. A UK national survey of clinicians' views on IUT in this particular context and their willingness to randomise patients within a definitive trial will help establish if sufficient clinicians are in equipoise. Qualitative interviews with a sub group of clinicians will help contextualise the responses. During the qualitative interviews with the women willingness to be randomised will also be explored.

Attempts to estimate sample size for a definitive trial were unproductive because of uncertainty in the distribution of the key variables. The pilot study aims to collect data on the primary outcome measure, the ICIQ-FLUTS combined symptom score, to calculate the sample size for a definitive trial.

The decision to proceed with the definitive trial as it is currently envisaged will only be made if all aspects of feasibility, including adequate patient recruitment and clinician engagement are established. The combination of quantitative methods (*e.g*. recruitment and retention rates, percentage of clinicians in clinical equipoise) and qualitative methods (*e.g*. an assessment of whether any identified barriers to recruitment and retention could be overcome by modifications to trial design or procedures) will inform the decision and ensure its effectiveness in achieving the research aim.

Currently there are two other on-going trials looking at the clinical utility of urodynamics in similar patient groups; the VaIUE trial (Value of Urodynamic Evaluation)[39] and VUSIS-2 trial (Value of Urodynamics prior to Stress Incontinence Surgery) [40]. Similar to INVESTIGATE-I, the ValUE study randomises women with a clinical diagnosis of SUI or stress predominant MUI to either no further assessment or IUT. In the VUSIS study all women undergo invasive urodynamic testing. Those with discordant clinical and urodynamic findings are randomised between surgical treatment as dictated by their clinical assessment and individualised treatment dictated by a combination of clinical and urodynamic results and therefore addresses a different clinical question. Both of these definitive trials use a non-inferiority design [41]. Whereas VUSIS does not define a non-inferiority margin VaIUE defines a margin of 11% which we consider somewhat high and a difference that might potentially influence the decisions of both clinicians and patients. The primary outcome of both is based on the Urogenital Distress Inventory (UDI) score at 12 months. Although our primary outcome is the ICIQ-FLUTS combined symptom score we have chosen to include the UDI as an additional secondary outcome. If we subsequently proceed to the definitive trial, INVESTIGATE-II, assuming the other studies complete recruitment and publish their results, this will allow easier comparison of results. While we are encouraged to see that others look on this topic as being an important clinical uncertainty, we remain of the opinion that a feasibility study is an important step before embarking on a definitive trial.

## Competing interests

PH - No current financial interests; previous chair of NICE Guideline Development Group (GDG) on urinary incontinence in women; previous member NETSCC-HTA Interventional Procedures Panel, and Clinical Evaluations and Trials Prioritisation Group; previous commercial research funding for trials of surgery for stress incontinence from *Gynecare *(1998-2003) and *Gyne Ideas *(now *Mpathy Medical*) (2001-2003). ML - No current financial interests; previous member of NICE GDG on urinary incontinence (UI) in women and on lower urinary tract symptoms in men; chair European Association of Urology guideline group on UI. CRC - Consultant and researcher for Astellas, Pfizer, Novartis, Allergan & Recordati; consultant for Ono & Xention. DGT - Consultancy work for Pfizer, Ethicon, Galen; research grants from Ethicon, Astellas. Chair of BSUG Research Committee. MM, EMcC, DH, MD, NA, BB, CB, LV, JS - None.

## Authors' contributions

PH - is the lead grant holder, he conceived the study, led on the protocol development, contributed to writing the manuscript, and approved the final version for publication. MM - wrote the paper and approved the final version for publication. EM, DH, MD, BB, ML, CRC, DGT, NA - are co-holders of the grant, contributed to protocol development and to writing the manuscript, and approved the final version for publication. CB, JS, LV - contributed to protocol development and to writing the manuscript, and approved the final version for publication.
